# RNA-induced Allosteric Coupling Drives Viral Capsid Assembly

**DOI:** 10.1103/prxlife.2.013012

**Published:** 2024-03-12

**Authors:** Sean Hamilton, Tushar Modi, Petr Šulc, S. Banu Ozkan

**Affiliations:** 1 Center for Biological Physics, Arizona State University, Tempe, Arizona 85281, USA; 2 School of Molecular Sciences and Center for Molecular Design and Biomimetics, The Biodesign Institute, Arizona State University, Tempe, Arizona 85281, USA; 3 School of Natural Sciences, Department of Bioscience, Technical University Munich, 85748 Garching, Germany

## Abstract

Understanding the mechanisms by which single-stranded RNA viruses regulate capsid assembly around their RNA genomes has become increasingly important for the development of both antiviral treatments and drug delivery systems. In this study, we investigate the effects of RNA-induced allostery in a single-stranded RNA virus—Levivirus bacteriophage MS2 assembly—using the computational methods of the Dynamic Flexibility Index and the Dynamic Coupling Index. We demonstrate that not only does asymmetric binding of RNA to a symmetric MS2 coat protein dimer increase the flexibility of the distant FG-loop, inducing a conformational change to an asymmetric dimer, but also RNA binding reorganizes long-distance communications, making all the other positions extremely sensitive to the fluctuation of the ordered FG-loop. Additionally, we find that a point mutation in the FG-loop, W82R, leads to the loss of this asymmetry in communications, likely being a leading cause for assembly-deficient dimers. Lastly, this dominant communication that enhances its dynamic coupling with all the distal positions is not only a property of the dimer but is also exhibited by all the observed capsid intermediates. This strong dynamic coupling allows for unidirectional signal transduction that drives the formation of the experimentally observed capsid intermediates and fully assembled capsid.

## I. INTRODUCTION

Single-stranded RNA (ssRNA) viruses package their genetic material into a protein capsid as part of their replication cycle [1–3]. Understanding the mechanism of the packaging has importance for the development of antiviral therapeutics, as well as for designing delivery applications in bionanotechnology. The main experimental source of information about viral packaging is the atomistic structures resolved through cryoEM or x-ray crystallography, which, however, only provide a static picture of the assembled virus. Several prior studies included coarse-grained simulations of capsid proteins aggregating around negatively charged ssRNA [4]. The pathways of assembly differ between viruses, and the proposed mechanisms include aggregation around a viral genome followed by gradual rearrangement, and sequential growth where upon a capsid binding to the viral genome, the subsequent growth is driven by interactions between domains of capsid proteins. Various experimental studies of assembly have also been explored in recent years, highlighting the importance of RNA-protein interactions in the self-assembly of virions [5,6]. A mechanism of RNA packaging signals has been proposed for MS2 and GA virions, where RNA binding promotes protein-protein interactions during the assembly process [7].

Researchers have extensively studied the *Levivirus* bacteriophage MS2, a positive-sense ssRNA virus that encodes only four proteins: maturation, lysis, replicase, and coat [8–10]. X-ray crystallography (PDB ID: 1ZDH [11]) has revealed that the MS2 genome is encapsulated by 180 coat proteins that form three quasiequivalent conformations arranged as 60 asymmetric A/B dimers and 30 symmetric C/C dimers. These dimers create a T=3 icosahedral shell with spherically symmetric threefold (pseudo-sixfold) and fivefold axes. The FG-loops (residues 66–82) of the symmetric and asymmetric dimers, which make crucial interdimer contacts at the threefold axis (FG-loops A and C) and at the fivefold axis (FG-loops B), have significant structural differences [11,12].

Upon binding to a 19-nt hairpin operator within the replicase gene, the β-sheet interface of the MS2 coat protein dimer acts as a translational repressor [13–15]. NMR experiments have shown that the coat protein dimer favors the symmetric structure in the absence of genomic RNA, whereas it favors the asymmetric structure when bound to the RNA-hairpin operator [16]. Self-assembly kinetics measurements indicate that the assembly process largely depends on the relative concentrations of coat protein and cognate RNA, with an asymmetric dimer bound to the hairpin operator acting as the nucleation site for capsid assembly [13,14,17,18]. The MS2 coat protein can bind to various nonoperator RNA-hairpins with high affinity, suggesting that the genome contains at least 59 other non-sequence-specific hairpins that are necessary for successful assembly [14,19,20]. Mass spectrometry has revealed multiple protein-RNA contacts within the fully assembled capsid, providing direct evidence that MS2 viral assembly is mediated via noncovalent interactions at RNA-binding regions, referred to as “packaging signals” [21–24].

In summary, all these prior works clearly support the critical role of RNA-induced allosteric regulation in MS2 assembly. Allosteric control is the ability of one part of a protein to control another part through changes in conformation upon ligand binding at the controller site, allowing for the integration of multiple signals and the coordination of complex biological processes [25–28]. Allostery plays a vital role in the cooperative assembly of the viral capsid [29]. Previously, the conformational change of the dimeric FG-loops, critical to capsid assembly, has been shown to be modulated by RNA binding [30,31]. While in the absence of RNA the two FG-loops in the unbound dimer are symmetrically coupled, RNA binding induces disorder in the distal of the FG-loop (i.e., the FG loop that is farther away from the RNA binding site, denoted as C*), leading to an asymmetric structure, necessary for virus assembly.

However, it has been shown that capsid assembly can be disrupted independently of repressor activity due to single point mutations in the FG-loop [15,16,32,33], despite the fact that single point mutation prevents neither RNA binding nor the shift in flexibility of the two FG loops. Thus, we hypothesize that RNA-induced allosteric regulation in capsid assembly goes beyond induced conformational change, but also orchestrates long-distance communication that enhances the cooperativity of binding [34,35]. For allostery to effectively regulate interactions, there must be one domain that controls a separate, usually distant, domain. To evaluate the long-distance communication and asymmetry in communications, we use here previously developed methods of the Dynamic Flexibility Index (DFI) and the Dynamic Coupling Index (DCI) to study the allosteric regulation of MS2 viral capsids induced by binding of its RNA genome. The DFI quantifies flexibility of respective protein residues by quantifying their displacement under an applied force using linear response theory [36]. DFI has been previously shown to identify residues important to protein function, as mutations introduced at rigid positions result in dysfunctional proteins [27,28]. DCI measures coupling between pairs of residues upon perturbation of one of them. Prior studies showed that high DCI scores between distant residues indicate long-range allosteric communication [27,28].

Our results show that, in the symmetric MS2 coat protein dimer, the two FG-loops act as highly flexible and highly coupled domains that communicate symmetrically. When the 19-nt hairpin RNA binds to its native site on one side of the β-sheet interface, the flexibility of the opposite FG-loop increases, and the symmetric coupling is broken. This induces a cascade that signals for the FG-loop to shift into the rigid conformational state found in the asymmetric dimer. The fluctuation of ordered FG-loop A influences the motion of disordered FG-loop B, as evidenced by high DFI in FG-loop A and high DCI in FG-loop B when FG-loop A is perturbed, demonstrating the concept of allostery. This controller-controlled dynamic allows for highly regulated communication and unidirectional signal transduction. The unidirectional signal transduction propagates to higher-order complexes seen in the threefold and fivefold ring intermediates during capsid assembly. Upon RNA binding, one FG-loop becomes rigid and acts as a hinge, initiating capsid assembly at the fivefold ring. The flexible FG-loop of the asymmetric dimer acts as a signal transducer that interacts with the flexible FG-loop of a symmetric dimer, promoting cooperativity and long-range communication. This communication creates an allosteric network in which the flexible FG-loop modulates the orientation of an incoming dimer, triggering a cascade that allows each newly associated dimer to become the controller of the growing complex. Lastly, in the full capsid, we see that the rigid FG-loops of the fivefold axis provide structural stability with their low coupling, and the flexible FG-loops of the threefold axis propagate signals to the next threefold axis, suggesting a potential mechanism for disassembly.

In summary, our results of the application of DFI and DCI to MS2 bacteriophage indicate a mechanism of packaging signals that induces sequential assembly of the viral capsid, highlighting how RNA binding modulates long-distance communications to drive assembly. The approach can also be applied to other RNA-protein complexes for identification of possible RNA-mediated allosteric regulation of proteins.

## II. RESULTS AND DISCUSSION

### A. DFI analysis of the MS2 wildtype coat protein dimers and full capsid

**FIG. 1.**
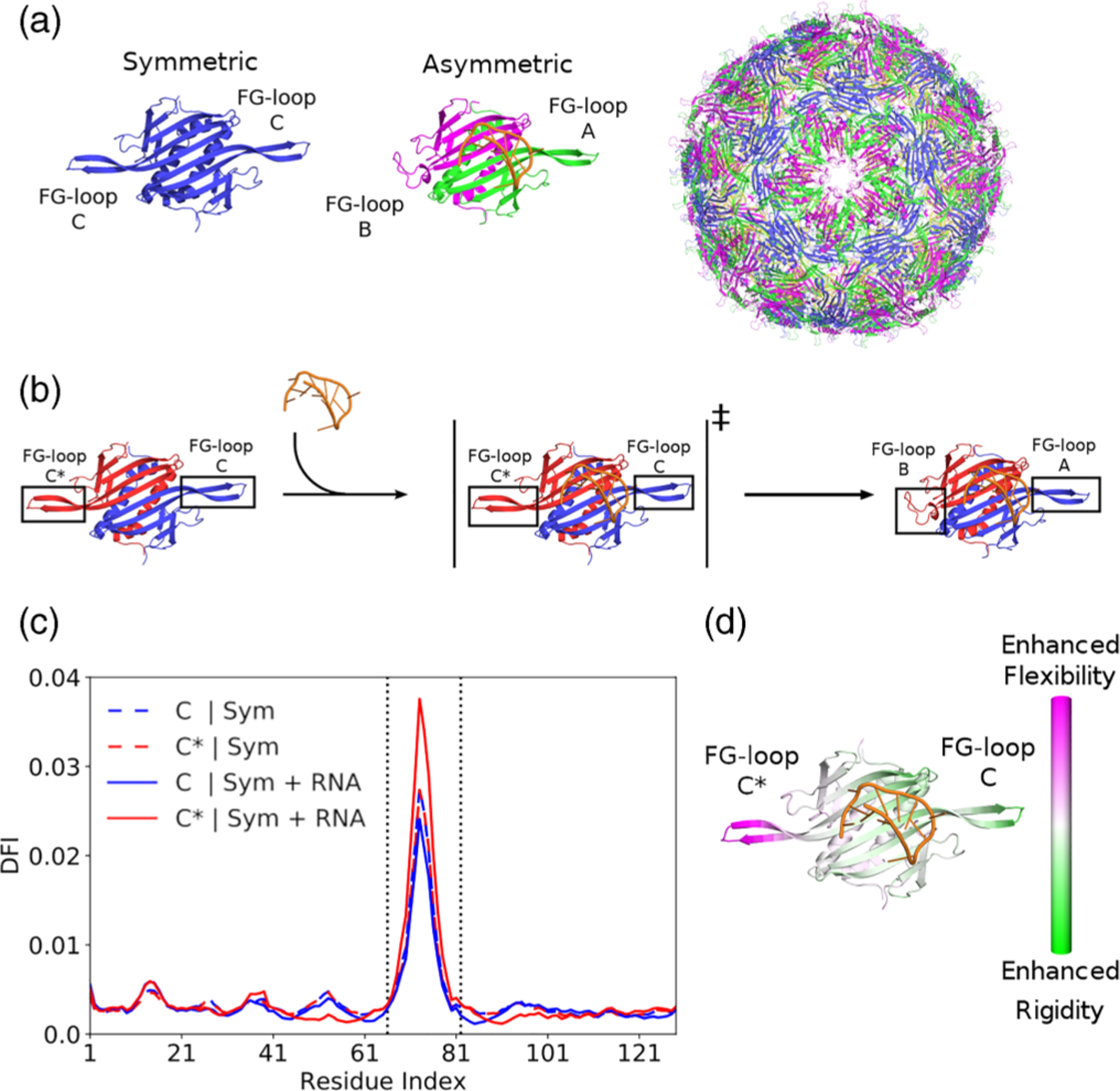
DFI profile and RNA-induced allostery in bacteriophage MS2 coat protein. (a) Cartoon structure of the MS2 capsid (PDB: 1ZDH) and its coat protein dimers. FG-loop B of the asymmetric dimers form the fivefold axis shown in the capsid on the left. FG-loops C and A alternate to form the threefold axes that surround the fivefold axis. (b) Cartoon diagram of the RNA-induced allosteric pathway for a single coat protein dimer (chain A and C in blue; chain B and C* in red). The symmetric dimer bound to RNA represents a transition state that induces a conformational change in the FG-loop farthest away from the binding site (FG-loop C*). (c) DFI profile for each of the coat protein monomers in the unbound symmetric dimer (dashed lines) and the bound symmetric dimer (solid lines). Between the dotted black lines are the FG-loop residues (66–82), which also have the highest dynamic flexibility. (d) Change in DFI from the unbound symmetric dimer to the bound symmetric dimer. RNA-binding enhances the flexibility of the FG-loop that is distal from the binding interface (FG-loop C*), indicating allosteric communication between the two domains.

Figure 1(a) shows a cartoon of the fully assembled MS2 capsid and its coat protein dimers. The two FG-loops of the symmetric dimer are equivalent, and therefore the one that goes through the conformational change will be denoted as C* throughout the paper. The RNA-binding pathway depicted in Fig. 1(b) drives the transition between two quasiequivalent conformers of the wildtype MS2 coat protein dimer. In the native state, an RNA hairpin binds to one side of the dimer’s β-sheet interface near FG-loop C but distant from FG-loop C*. Binding of RNA triggers a conformational change in FG-loop C*, resulting in an asymmetric A/B dimer [see Fig. 1(b)]. RNA binding induces long-distance effects on FG-loop C*, thus allosterically modulating the structural disorder transition. The symmetric wildtype dimer’s DFI profile before and after RNA-binding is presented in Fig. 1(c) (see Fig. S1 of the Supplemental Material [37] for DFI analysis of the symmetric mutant dimer and superimposition with the symmetric wildtype dimer). Without RNA, both FG-loops exhibit equal flexibility, reflecting the dimer’s symmetry. However, immediately after RNA-binding, the two FG-loops’ DFI symmetry is disrupted as the transition to the asymmetric dimer occurs. Because the average distance between FG-loop C* and the RNA-binding sites is 34.1 Å (calculated in PyMOL), the increased dynamic flexibility of FG-loop C* [Fig. 1(d)] indicates the presence of RNA-induced allostery. This observation is consistent with a prior all-atom normal-mode analysis of the same system [30,31].

**FIG. 2.**
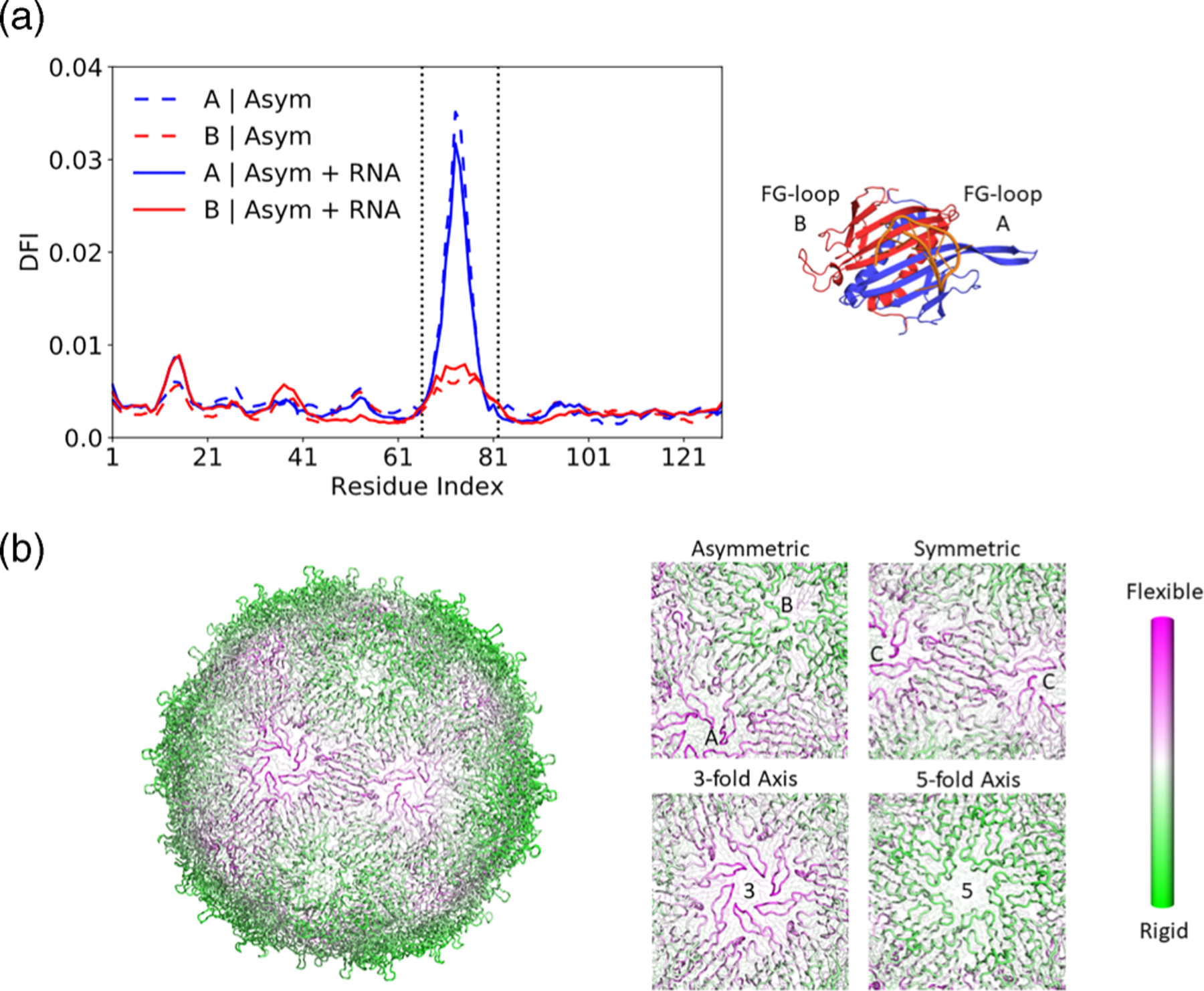
DFI profiles of asymmetric dimer and fully assembled capsid. (a) DFI profile for each of the coat protein monomers in the unbound asymmetric dimer (dashed lines) and the bound asymmetric dimer (solid lines). One of the flexible FG-loops has now become rigid, with little to no change upon binding of RNA. (b) Color-coded DFI profile of the fully assembled *T* = 3 capsid in the absence of RNA. A closeup is shown of an asymmetric dimer, a symmetric dimer, and the threefold/fivefold axes. Even in the fully assembled capsid, the ordered FG-loop A/C retains its flexibility while the disordered FG-loop B remains rigid.

The DFI profile for the asymmetric dimer is shown in Fig. 2(a). Remarkably, following the conformational change, the FG-loop B undergoes a significant reduction in flexibility, even in the absence of RNA. This can be attributed to the higher number of close contacts experienced by FG-loop B. To gauge the universality of this flexibility change across dimers, we extended our analysis to encompass a fully assembled MS2 capsid, as depicted in Fig. 2(b). Intriguingly, while the FG-loops of symmetric dimers maintain their high flexibility, one of the FG-loops in each asymmetric dimer remains rigid. This observation underscores that the dynamic characteristics of individual dimers persist even within the context of higher-order structures. Based on the flexibility profiles of the whole capsid, it is conceivable that the rigid fivefold axes serve as pivotal points that uphold the integrity of the assembled capsid, while the flexible threefold axes offer a mechanism for disassembly upon entry into the target cell.

### B. DCI analysis of wildtype coat protein dimers

**FIG. 3.**
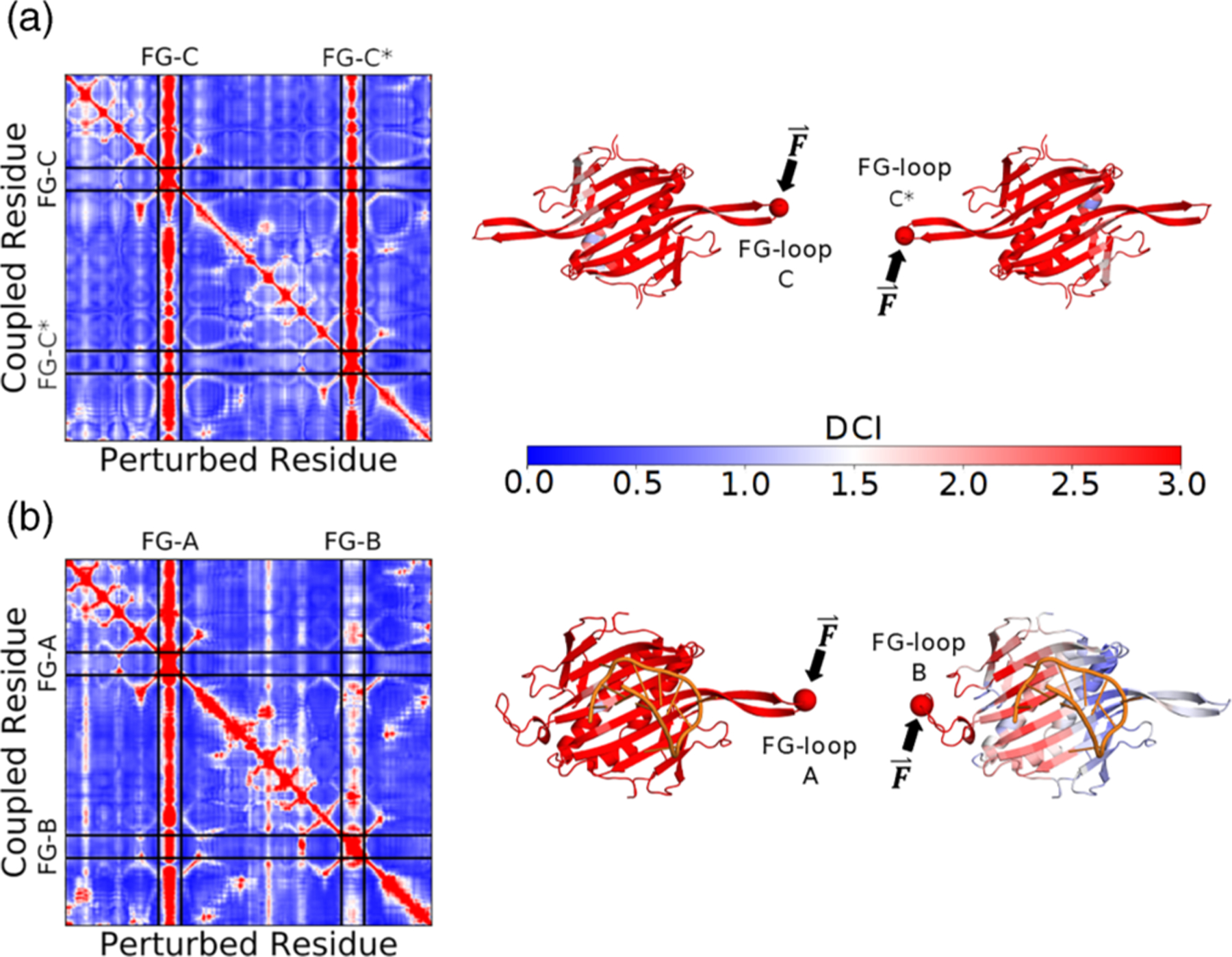
DCI profiles for the unbound symmetric and bound asymmetric wildtype MS2 coat protein dimers. (a) DCI profile for the unbound symmetric dimer. The FG-loops are symmetrically coupled, where a perturbation at one end of the dimer produces a strong response at the other end. (b) DCI profile for the bound asymmetric dimer. The symmetric coupling of the two FG-loops is broken, such that FG-loop A now drives the communication to FG-loop B. All cartoon structures are colored with DCI for perturbations at residue 74 (shown as a sphere).

To further investigate how the RNA-induced conformational change facilitates successful capsid assembly, we examined the DCI profiles of the wildtype unbound symmetric dimer and the wildtype bound asymmetric dimer, as shown in Fig. 3 (see Fig. S2 for DCI analysis of the mutant dimer). In the unbound symmetric dimer [Fig. 3(a)], the FG-loops and the binding interface are strongly coupled to each other. Conversely, in the bound asymmetric dimer [Fig. 3(b)], while FG-loops A/B are still highly coupled to the binding interface, only FG-loop A (in the flexible conformation) is highly coupled to FG-loop B (in the rigid conformation). Hence, the RNA-induced conformational change results in unidirectional asymmetric communication between the FG-loops, which propagates from FG-loop A to FG-loop B. These findings suggest that the flexible FG-loop A serves as the primary communicator during the initiation of capsid assembly. This asymmetry in dynamic coupling creates a causality relationship [35,38,39] and has a critical impact on the cooperative assembly of the MS2 capsid. When the FG-loop A of an asymmetric dimer forms interdimer contacts with the FG-loop C of a symmetric dimer, it results in the long-range coupling of FG-loop A with the free FG-loop C farthest away from the interaction point.

The W82R mutant dimer (PDB: 1MSC), which binds to RNA and exhibits distinct flexibility profiles when bound to RNA (see Fig. S1), lacks the capability for capsid assembly [31,32]. This presents a challenge in comprehending why a mutant with similar biophysical properties to the wildtype dimer cannot undergo a cooperative capsid assembly process. Interestingly, the asymmetry in dynamic coupling of the two FG-loops is not observed in the W82R mutant dimer (see Fig. S2), particularly having a negligible effect on the dynamic coupling between FG-loops. These observations may align with a prior study suggesting that the W82R mutation restricts the conformational and free-energy landscape of the symmetric dimer [32,33], possibly due to the replacement of a large hydrophobic amino acid (Trp) with a smaller hydrophilic one (Arg). This may be what prevents the FG-loop C* (the loop far from the RNA binding) from becoming susceptible to the motion of the FG-loop C (i.e., asymmetry in communications and thereby impacting the cooperative assembly). Thus, we conclude that the RNA-induced asymmetry in dynamic coupling of the FG-loops is crucial for the proper self-assembly of the MS2 capsid.

### C. DCI analysis of intermediates and the full MS2 capsid

**FIG. 4.**
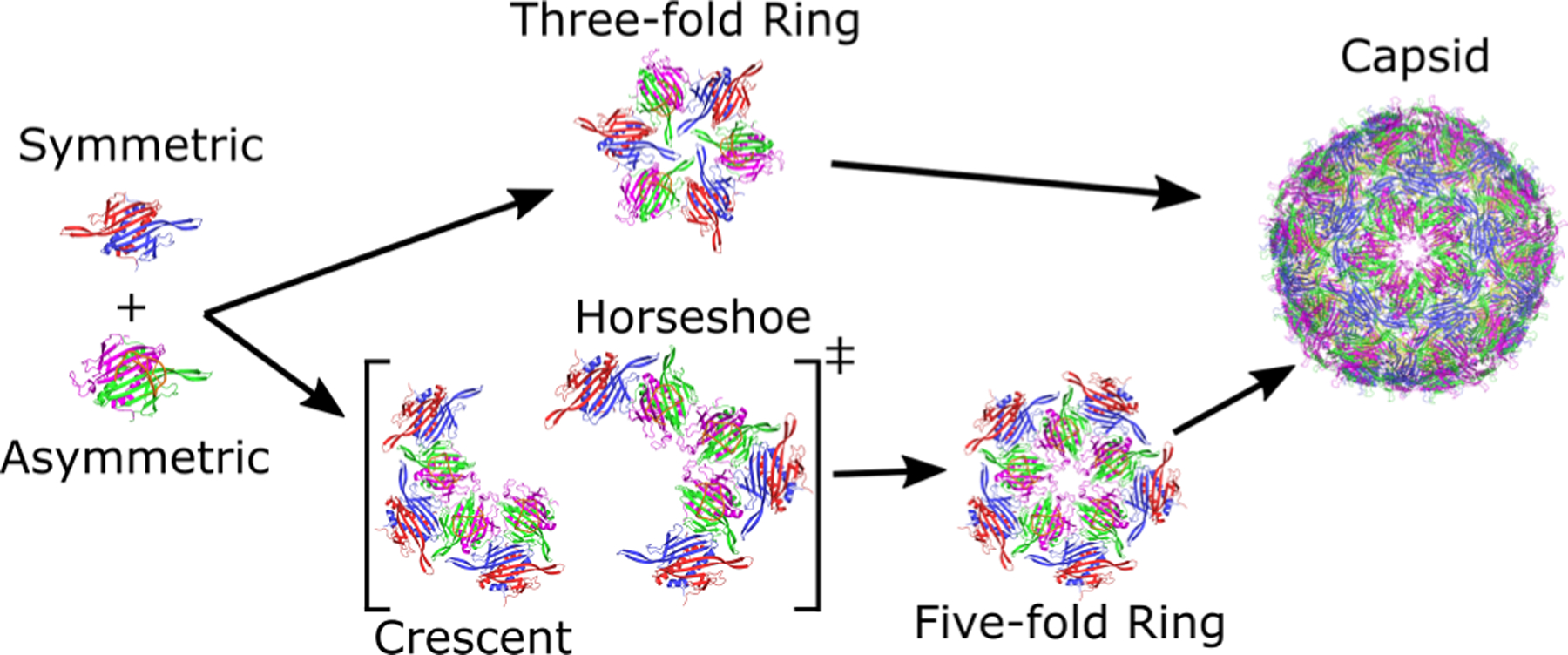
The proposed assembly pathway for bacteriophage MS2 capsid. Higher-order capsid intermediates are formed by the alternating 1:1 association of asymmetric-symmetric dimers. Threefold rings and fivefold rings form independently, with an RNA-bound asymmetric dimer acting as the nucleation site of capsid assembly. Crescent and horseshoe conformations are two possible intermediates that arise during the formation of the fivefold ring structure. In the full *T* = 3 capsid, five threefold rings converge at one fivefold ring.

There are several higher-order structures proposed for the assembly pathway of the bacteriophage MS2 capsid (Fig. 4). Although detecting protein complex intermediates can be challenging due to their short lifespan, two significant intermediates, namely the threefold and fivefold rings, have been identified on the MS2 capsid assembly pathway via mass spectrometry [40–42]. While crescent and horseshoe structures have not been experimentally detected, theoretical evidence suggests that they are two probable structures on the pathway to the fivefold ring (see [40,42]).

The successful self-assembly of biomolecules requires long-range cooperativity, which we hypothesize will be reflected in the intermediate structures along the pathway to capsid assembly. Specifically, we predict that these intermediates should exhibit asymmetric high coupling in the same domains as individual coat protein dimers, namely the motion of flexible FG-loops. Indeed, earlier experimental and computational studies also support the significance of the ordered FG-loop. When the symmetric dimer associates with the asymmetric dimer through interactions involving ordered, flexible FG-loops, it enables the next asymmetric unit to associate through interactions with disordered FG-loops, resulting in the formation of a fivefold ring intermediate. This aligns with proposed assembly pathways that promote an alternating association of asymmetric-to-symmetric dimers [40,41]. Therefore, we hypothesize that the observed highly dynamic coupling of all the positions with the ordered FG-loop should manifest across different lengthscales and emerge as a recurring pattern observed in intermediates.

**FIG. 5.**
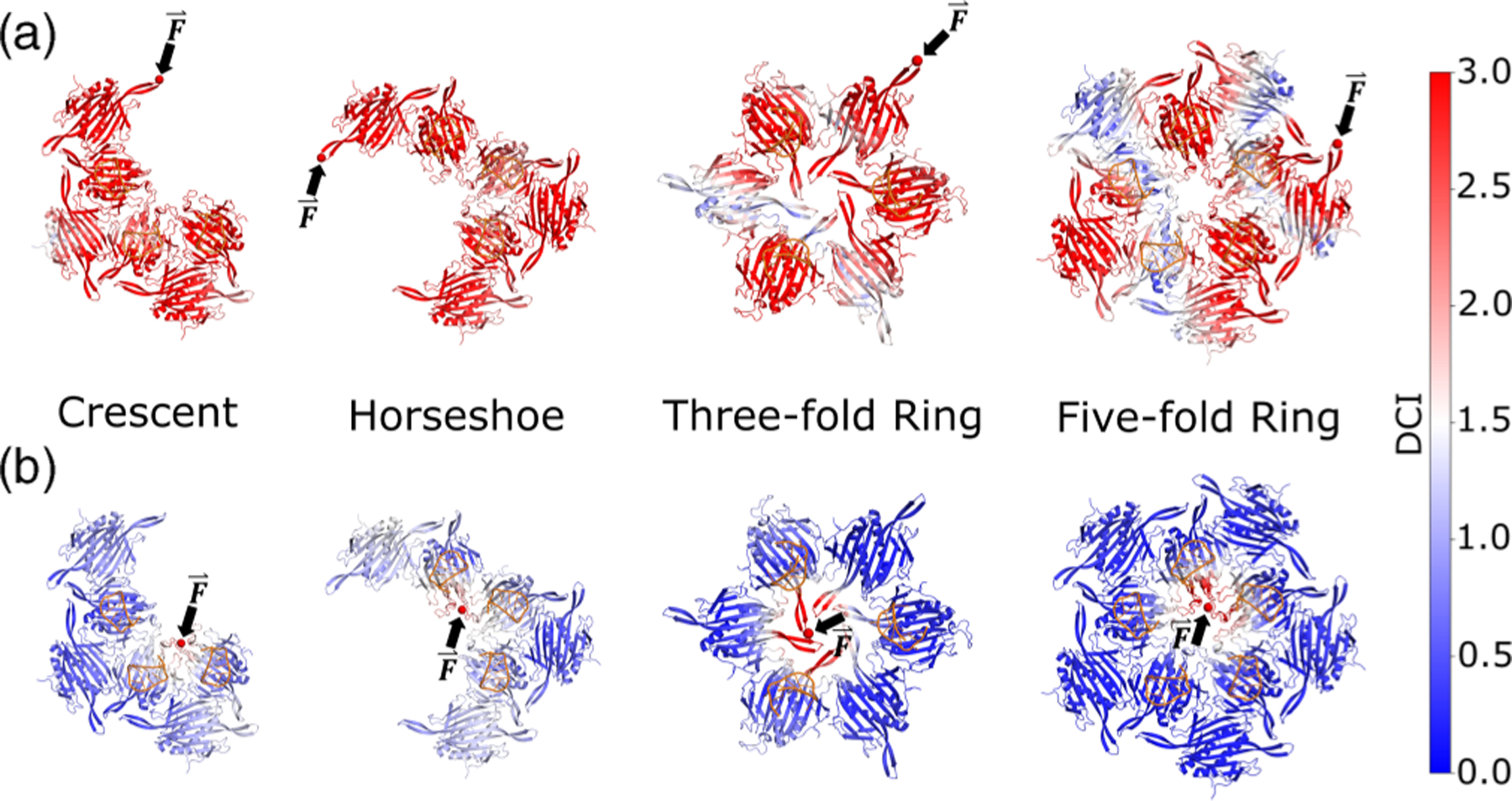
DCI for perturbations at FG-loop residue 74 in intermediate capsid structures. (a) DCI spectrum for a flexible FG-loop on an outer dimer of the intermediate. These perturbations have rotational symmetry (see Fig. S3) and are highly coupled, indicating long-range communication among these FG-loops during the formation of the threefold and fivefold axes seen in the full capsid. (b) DCI spectrum for an FG-loop on an inner dimer of the intermediate. In contrast to the outer FG-loops, perturbations of inner FG-loops have low dynamic coupling beyond the localized region at the center of the ring, suggesting structural stability of the FG-loop contacts at the fivefold and threefold axes during assembly.

To test this hypothesis, we analyzed the DCI profiles of intermediate structures when an outer or inner FG-loop is perturbed (Fig. 5). We observed that perturbations at outer FG-loops, which have few to no interdimer contacts, exhibit high coupling that cascades throughout the entire structure [Fig. 5(a)]. Additionally, for the threefold ring and the fivefold ring, perturbations at any of the three or five outer FG-loops, respectively, result in a strong response everywhere in the structure, reflecting their rotational symmetry (see Fig. S3). In contrast, perturbations at inner FG-loops, where interdimer contacts are fully saturated, do not propagate beyond their immediate neighbors, indicating a region of high stability at the center of the threefold and fivefold axes during assembly.

The effectiveness of the fivefold axis as a nucleation site for capsid assembly is due to the low DFI and DCI of its comprising FG-loops. These FG-loops act as a stable hinge during the formation of new interdimer contacts on the pathway from crescent/horseshoe to the fivefold ring, since they are weakly coupled to the rest of the structure. Additionally, the outer FG-loops of each intermediate are flexible and highly coupled, providing the necessary mobility to guide newly associated dimers into the correct orientation and minimizing the number of exposed flexible FG-loops. Consequently, assembly occurs spontaneously and more efficiently around the fivefold and threefold axes, and the assembly process continues until no more dimers can fit into the fully assembled, spherically symmetric capsid.

**FIG. 6.**
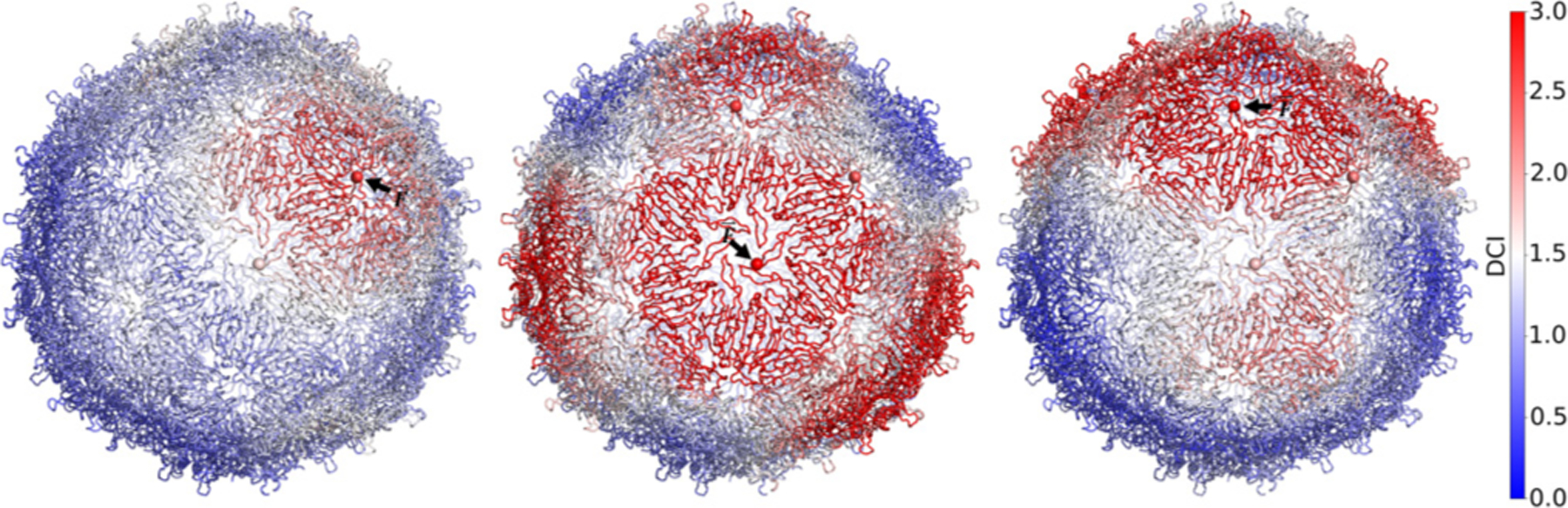
DCI profile of a fully assembled capsid upon perturbation of residue 74 in various FG-loops. Perturbations at the threefold axis propagate further than perturbations at the fivefold axis. Overall, the asymmetric coupling between the two FG-loop conformers is still observed. Perturbations at the flexible threefold axis propagate to the neighboring threefold axes, while perturbations at the rigid fivefold axis are highly localized. Thus, the flexible FG-loops continue to be the primary communicators.

Figure 6 shows the color-coded DCI profile for the fully assembled capsid. In contrast to the threefold ring intermediate—where perturbations at the center were highly localized—we see that perturbations at the center of the threefold axis propagate further than those at the center of the fivefold axis. This asymmetry in coupling between the two axes reflects the importance of the two FG-loop conformations. Once assembly has completed, the fivefold axes continue to provide the structural stability to the newly formed capsid, whereas the threefold axes are highly flexible and highly coupled, a potential mechanism by which the capsid may disassemble. Thus, we propose that RNA is an important allosteric regulator in MS2 capsid assembly because it gives rise to the rigid FG-loops that comprise the stable fivefold axes.

## III. CONCLUSION

Self-assembling viruses represent the most basic biological system that is capable of constructing itself, yet still relies on host organisms for replication. The interaction between proteins and RNA, which are ubiquitous in ssRNA viruses, is of great interest to molecular biologists and researchers seeking to develop new tools for gene therapy and drug delivery [43]. While DFI and DCI have proven effective in measuring the dynamic interactions between a small coat protein and RNA in the case of MS2, further investigations are needed to understand the mechanism in other viral species. For instance, although closely related to MS2, the FG-loops of *Levivirus* bacteriophage PP7 do not undergo conformational changes, despite similar RNA packaging signals and regulatory activity in capsid assembly [44,45]. Experimental research has also shown that brome mosaic virus (BMV) and cowpea chlorotic mottle virus (CCMV)—which are both plant viruses—also self-assemble via RNA-mediated signaling, suggesting that the mechanism discussed here may be general to all viruses [5,6]. Moreover, the recent discovery of previously unknown variability in MS2 capsids [46] raises questions about whether the protein-RNA interactions discussed here are specific enough to support the formation of T=3 symmetry over unconventional T=4 or hybrid T=3/T=4 symmetries. Finally, the application of DFI and DCI to other viral proteins and even larger RNA-protein systems, such as ribosomes, spliceosomes, CRISPR-Cas9, and other capsids, has the potential to reveal a general allosteric mechanism among nucleoprotein complexes that can be guiding their function as well as mediate a particular directed assembly pathway, and they will be the subject of our future investigations. The DCI and DFI methods are also much less computationally demanding than molecular-dynamics simulations, allowing an efficient method to analyze allosteric interactions induced by RNA in protein-RNA complexes, thus presenting an effective tool for studies of dynamic couplings in such structures, and effects of various mutations on their function. However, RNA viruses can employ different strategies to assemble their capsids [2], and the effects of nonspecific charge interactions between RNA and capsid proteins, as well as the roles of capsid protein flexibility on the viral genome packaging and capsid assembly, have been theoretically investigated [47–49]. It is quite likely that the RNA-induced allostery can be combined with these effects as part of the evolved strategy to efficiently package viral genomes. The approach to study RNA-induced allostery that we proposed here can be used to construct and parametrize coarse-grained models of viral shells [4]. Molecular dynamics of such models can then be used to investigate virus capsid assembly pathways, and specifically study assembly kinetics with and without allosteric RNA-triggered binding and its effect on assembly yield. Such an effort will be the subject of our future studies, using our recently introduced allosteric patchy particle model [50].

## IV. METHODS

### A. Dynamic flexibility index and dynamic coupling index

The Elastic Network Model (ENM) is a useful tool for studying protein conformational space. It represents a polymer, such as an amino acid or nucleotide sequence, as a network of nodes connected by elastic harmonic springs. ENMs have been applied to proteins in the absence of RNA, using either all-atom or coarse-grained models of a single atom per residue, depending on the specific application [51]. In recent years, the ENM has been extended and applied to RNA structures [52]. However, due to the higher flexibility of RNA compared to proteins, RNA molecules are best modeled as coarse-grained networks using more than one atom per nucleotide [53,54]. For the analysis of MS2 capsid with its cognate RNA, we used coarse-grained models comprised of alpha carbons (CA) for the viral coat protein, and the phosphate/ribose sugar backbone atoms (P, C, and O) for the RNA-hairpin, ignoring the nitrogenous bases to avoid any sequence-specific interactions. For a comparison of coarse-grained RNA models, see Fig. S4.

ENMs allow for the measurement of protein conformational dynamics at the amino acid level by simulating perturbations and measuring the response of each residue. The magnitude of fluctuation in response to random forces is directly proportional to the elastic energy of normal modes in the spring system, indicating functionally important allosteric positions [27,28]. In this paper, two metrics—the Dynamic Flexibility Index (DFI) and the Dynamic Coupling Index (DCI)—are used to analyze structural flexibility and long-range signaling between nodes in a network. These metrics introduce Brownian forces $\vec{F}$ at each residue and calculate the response vector $\Delta \vec{R}$ using linear response theory (LRT), as shown in the equation

(1) $${\left[\Delta \vec{R}\right]}_{3N\times 1}={\left[H\right]}_{3N\times 3N}^{-1}{\left[\vec{F}\right]}_{3N\times 1},$$

where H is the 3N×3N Hessian matrix composed of the second derivatives of the harmonic ENM potential (scaled by $R^{-6}$ for each residue pair) with respect to the components of the 3N Cartesian position vectors. The Brownian forces simulate the chaotic environment within a cell, allowing for responses to be measured in a way that is most consistent with a naturally occurring system [27,28].

From the response vectors, we construct a perturbation matrix A of the form

(2) $$A_{N\times N}=\left[\begin{array}{ccc} {\left|\Delta {\vec{R}}^{1}\right|}_{1} & \cdots & {\left|\Delta {\vec{R}}^{N}\right|}_{1}\\ \vdots & \ddots & \vdots \\ {\left|\Delta {\vec{R}}^{1}\right|}_{N} & \cdots & {\left|\Delta {\vec{R}}^{N}\right|}_{N} \end{array}\right]$$

where ${\left|\Delta {\vec{R}}^{j}\right|}_{i}$ is the magnitude of the fluctuation of residue i when residue j is perturbed. Subsequently, the DFI value of residue i is given by the net fluctuation of residue i relative to the net displacement of the entire protein:

(3) $${\text{DFI}}_{i}=\frac{\sum_{j=1}^{N}{\left|\Delta {\vec{R}}^{j}\right|}_{i}}{\sum_{i=1}^{N}\sum_{j=1}^{N}{\left|\Delta {\vec{R}}^{j}\right|}_{i}}.$$

DFI is a metric that quantifies the relative flexibility of a residue, where higher values indicate more flexible positions that are functionally important (such as catalytic sites), and lower values indicate rigid positions that provide the structural integrity necessary to transmit perturbations to other residues. Computational studies using DFI have shown that point mutations at rigid positions (residues with low DFI) result in dysfunctional proteins, suggesting that these residues must be conserved across structural homologs with comparable function [27,28]. Thus, rigid residues act as hinges (like joints/pivots) in the global motion of the protein and are sequence-specific, while flexible (high DFI) residues provide the mobility necessary for dynamical processes such as catalysis, signal transduction, and conformational changes [27,28]. DFI has been used extensively to analyze the evolutionary history, structural stability, and dynamic allostery of various protein systems [55–58].

Similarly, the DCI value of residue i is given by the fluctuation of residue i in response to perturbations at a single residue j relative to the average response of residue i due to perturbations at all other residues:

(4) $${\text{DCI}}_{i}=\frac{{\left|\Delta {\vec{R}}^{j}\right|}_{i}}{\sum_{j=1}^{N}{\left|\Delta {\vec{R}}^{j}\right|}_{i}/N}.$$

DCI is a measure of the coupling between residue i and residue j upon perturbation at residue j. If the distance between residues i and j is significant, and binding of a ligand (such as hairpin RNA) increases their DCI, it suggests the presence of allosteric regulation between the sites. Previous studies on enzymes have demonstrated that certain residues distant from the active site exhibit high DCI, indicating long-range communication between functionally important residues and the allosteric sites that regulate activity [27,28]. DCI has also been used to study the role of distal sites in allosteric regulation in enzyme function and novel viral protein design [34,35,59,60]. Thus, DFI and DCI are powerful tools for studying protein-RNA interactions and their dynamics.

The MS2 coat protein dimer is only 252 residues, so calculating the inverse Hessian is trivial and computationally inexpensive. However, the full capsid is composed of 90 dimers (22 680 residues total). This makes the Hessian very large (68 040 × 68 040) and expensive to store in memory (80 GB for float32), and the eigenvalue decomposition is consequently impossible. Therefore, we used ARPACK [61], which is designed to compute a set of eigenvalues and corresponding eigenvectors numerically for a sparse matrix such as the Hessian. It uses a method called the Implicitly Restarted Lanczos Method [62] to recursively estimate the largest 20 eigenvalues and corresponding eigenvectors within the lowest possible tolerance (based on machine precision). These are then used to estimate the inverse of the Hessian matrix.

Amino acid and nucleotide coordinates for the wildtype coat protein dimers (symmetric, asymmetric, crescent, horseshoe, threefold ring, and fivefold ring) with and without RNA were generated from PDB: 1ZDH and exported via PyMOL. The mutant dimer (PDB: 1MSC) with docked wildtype RNA was generated from structure superposition on a wildtype bound asymmetric dimer using the cealign algorithm and exported via PyMOL. DFI and DCI were then calculated and plotted in Python JupyterLab using NUMPY, PANDAS, and MATPLOTLIB packages.

## Supplementary Material

supplemetary Figures
