# Supplementary material for: RNA-induced Allosteric Coupling Drives Viral Capsid Assembly
Source: PRX Life. Author manuscript; Available in PMC 2025 Aug 16. (PMC12356212; doi:10.1103/prxlife.2.013012)
Supplement: supplemetary Figures [file NIHMS2093707-supplement-supplemetary_Figures.pdf]

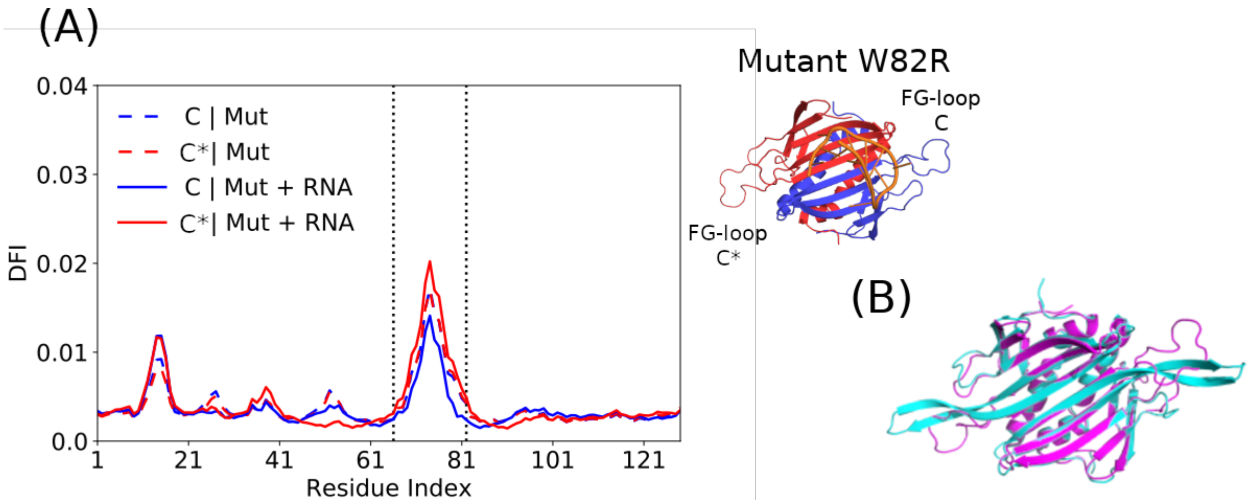

Figure S1. **(A) DFI profile for the symmetric mutant W82R coat protein dimer and (B) structural alignment to widltype symmetric dimer.** This mutant dimer is deficient in capsid assembly. When compared to the symmetric wildtype dimer, the mutant's change in DFI upon binding of RNA is not as significant, however the increase in flexibility of FG-loop C* when RNA is bound signifies the presence of RNA-induced allostery. The symmetric wildtype and mutant dimers have an RMSD of 3.1 Angstroms (calculated in PyMOL).

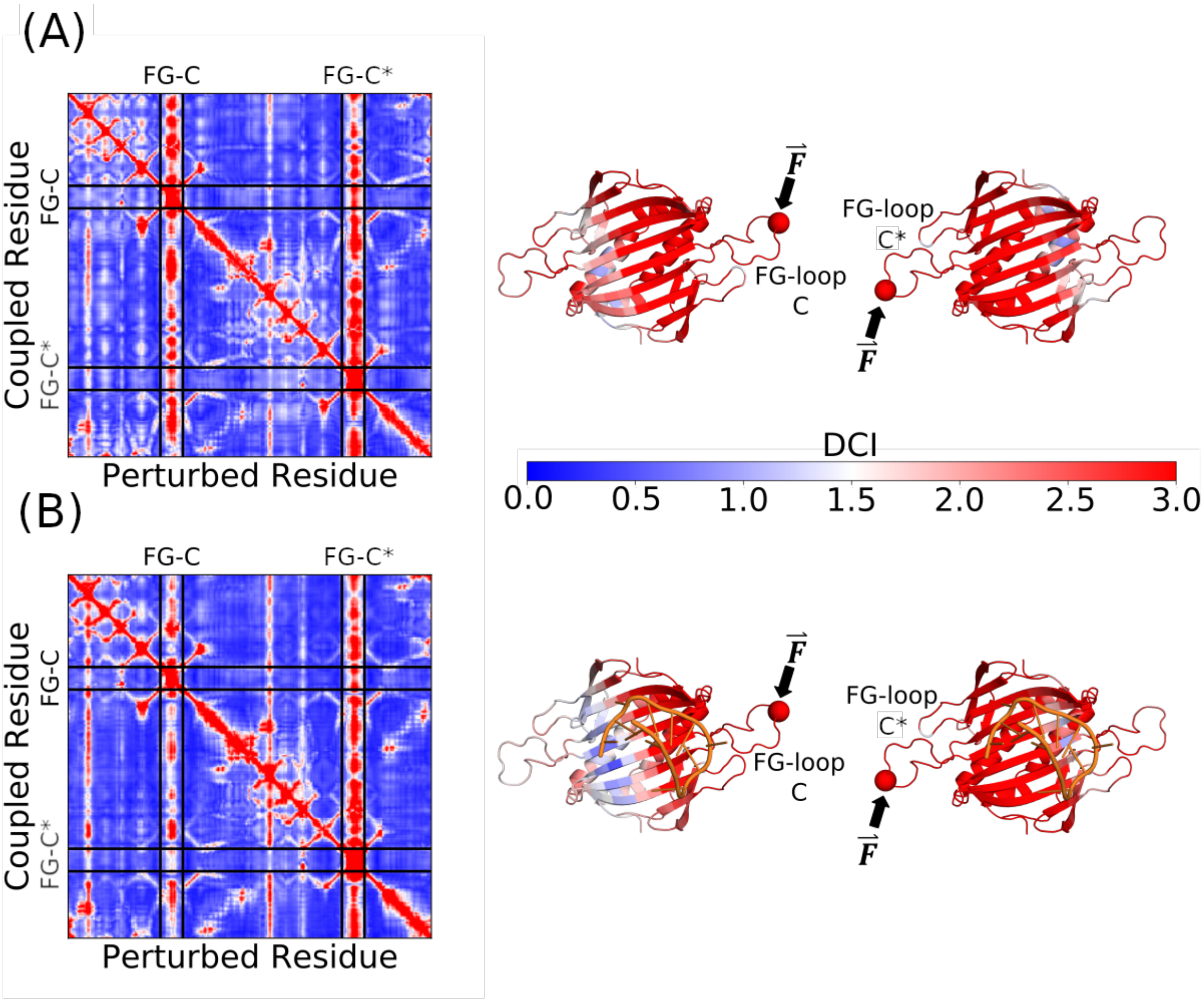

Figure S2. **DCI profiles for the unbound and bound mutant W82R coat protein dimer.** (A) DCI profile for the unbound mutant dimer. When compared with the wildtype dimer, the FG-loops are symmetrically coupled. (B) DCI profile for the bound mutant dimer. The dynamic coupling remains relatively the same as the unbound mutant dimer, suggesting a lack of RNA-induced asymmetric coupling. All cartoon structures are colored with DCI for perturbations at residue 74 (shown as a sphere).

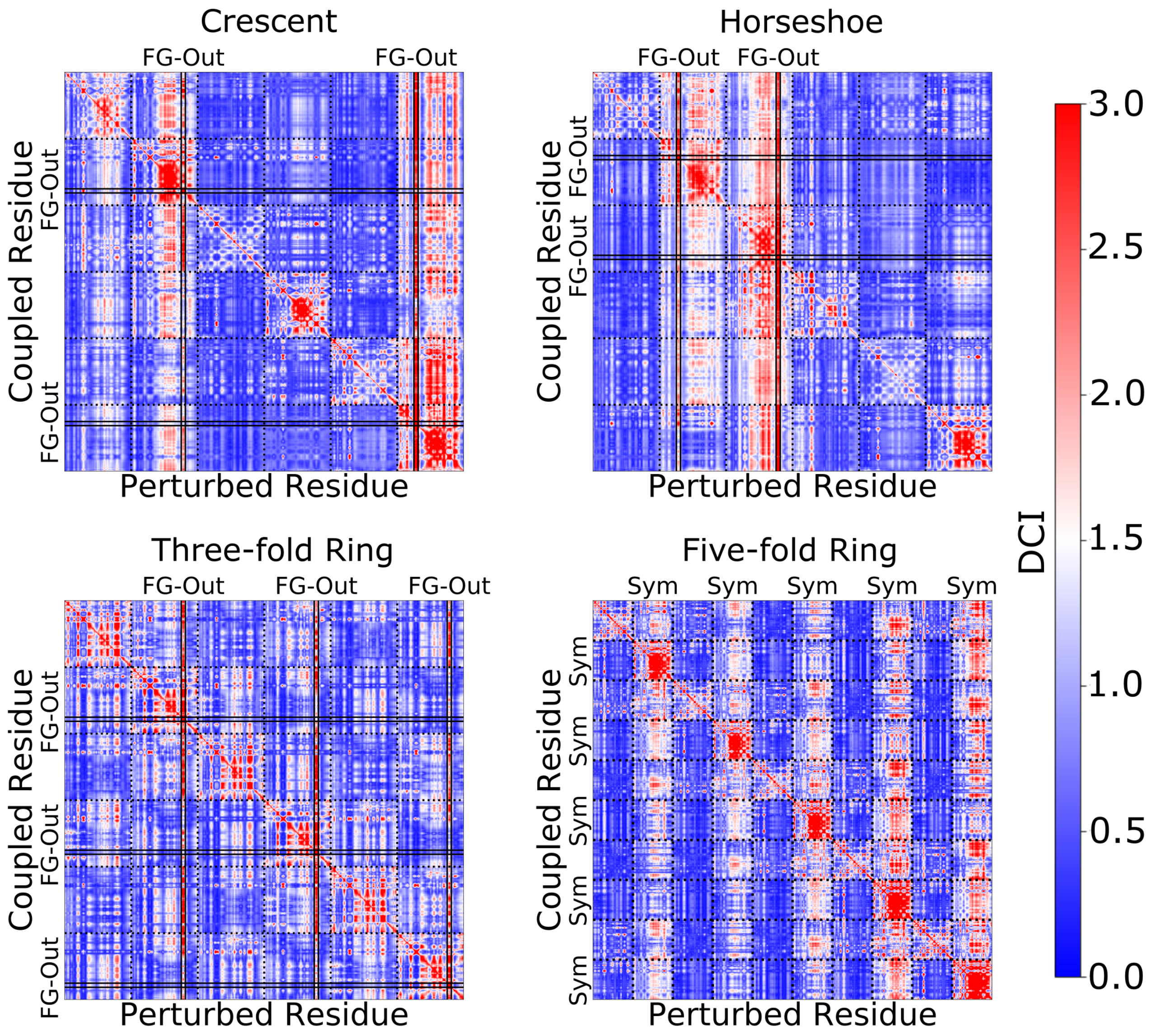

Figure S3. **DCI profiles for the proposed intermediate capsid structures.** The crescent and horseshoe structures each have two bands of high DCI that correspond to the two outer FG-loops that have no contacts to any other FG-loop. Despite the large distance between these two FG-loops, they are still highly coupled to each other. For the three-fold and five-fold rings, there are three and five bands of high DCI, respectively, corresponding to the symmetric dimers with flexible FG-loops furthest away from the center of the axis. These FG-loops are where contacts with the neighboring five-fold and three-fold rings occur. The long-range coupling ensures that proper capsid symmetry is achieved while maintaining the stability of the FG-loops that interact at these axes.

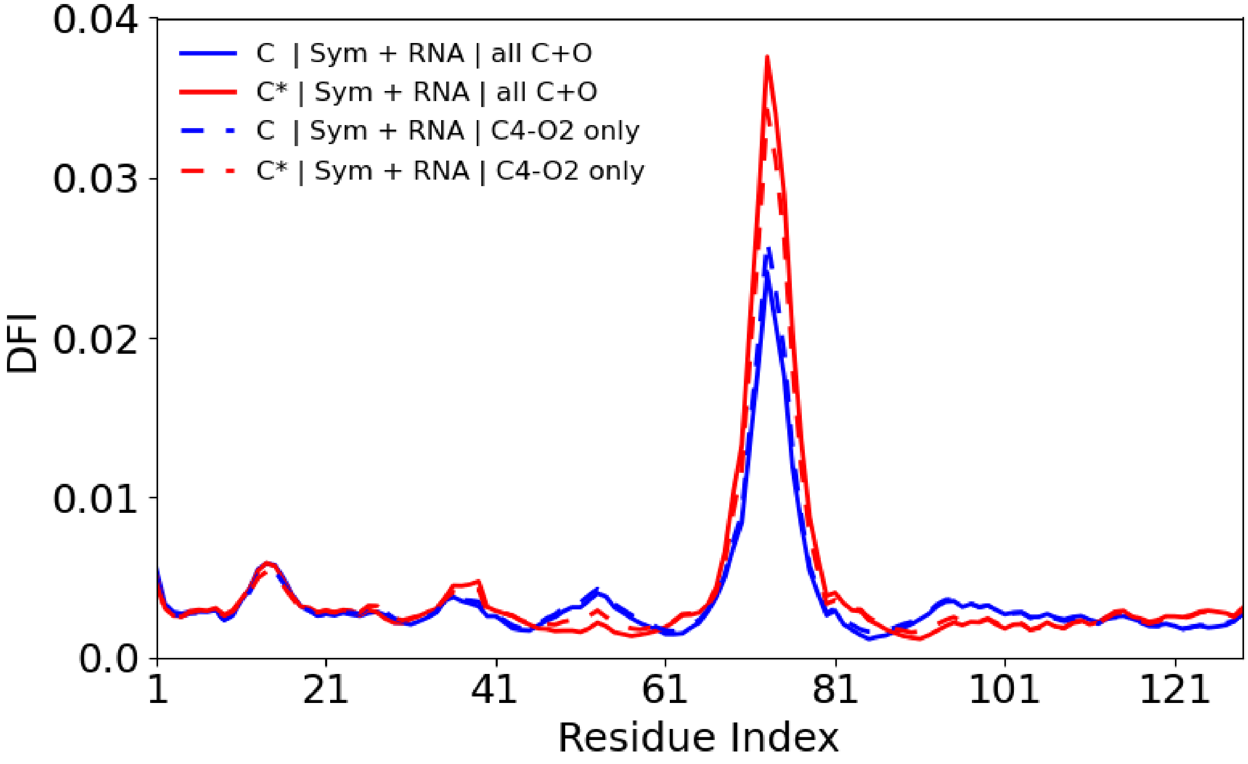

Figure S4. **Comparison of two different coarse-grained RNA models and their impact on DFI of the bound symmetric wildtype dimer.** The model chosen for the paper (solid lines) includes the phosphorus and all carbon and oxygen atoms of the RNA backbone, whereas another model (dashed lines) includes the phosphorus and only the C4 and O2 atoms of the ribose sugar. There is no significant difference between the two models when detecting RNA-induced allostery in MS2.
